# Recurrent Clostridium difficile Infections in a Patient With Ulcerative Colitis: A Case Report

**DOI:** 10.7759/cureus.95130

**Published:** 2025-10-22

**Authors:** Osama Nadeem, Muhammad Soban Imran, Nasir Siddique

**Affiliations:** 1 General Medicine, North Manchester General Hospital, Manchester, GBR; 2 General Medicine, Manchester Royal Infirmary, Manchester, GBR

**Keywords:** antibiotic-associated diarrhea, clostridium difficile infection treatment, elderly patients, inflammatory bowel disease, mesalazine, recurrent cdi, ulcerative colıtıs

## Abstract

*Clostridium difficile* infection (CDI) poses a substantial clinical challenge, especially in patients with inflammatory bowel disease (IBD), particularly ulcerative colitis (UC). Patients with UC are at greater risk of CDI and tend to experience a more severe disease course and higher rates of recurrence than the general population. We report a case of an elderly man in his early 90s with a long-standing history of UC treated with mesalazine, who had a prior hospitalization for hyponatremia and a history of CDI. During his most recent four-month hospitalization, he developed three separate episodes of CDI, confirmed by stool PCR and toxin assays. Despite treatment with vancomycin, metronidazole, and fidaxomicin in succession, he experienced recurrent episodes of CDI that ultimately progressed to septic shock and death. This case highlights the nature of recurrent CDI in this patient population and the complexity and increased morbidity associated with its management in elderly patients with UC. It underscores the importance of careful evaluation of underlying risk factors, judicious antibiotic use, and consideration of alternative treatment modalities, such as faecal microbiota transplantation (FMT), for the prevention of recurrent CDI.

## Introduction

*Clostridioides difficile* infection (CDI) is defined as a symptomatic infection of the large intestine caused by the anaerobic, spore-forming, gram-positive bacterium *C. difficile*. Recurrent CDI is defined as an episode of CDI occurring within eight weeks of a previous episode. CDI is widely recognized as the leading cause of antibiotic-associated diarrhoea, with increasing frequency and severity worldwide [1,2]. Inflammatory bowel disease (IBD), particularly ulcerative colitis (UC), occurs in the setting of a heightened risk of CDI due to alterations in the gut microbiota and impaired intestinal immunity [1,3]. CDI can negatively impact the clinical course of UC, leading to increased flares, hospitalizations, morbidity, and mortality [1,2]. The elderly with UC may be particularly at risk for CDI due to age-related changes in immunity, greater exposure to antibiotics, and the presence of comorbidities [1,3]. Recurrence remains a significant concern despite the use of recommended antimicrobial treatments, including vancomycin, metronidazole, and fidaxomicin [2,4]. New therapies have shown promise, such as faecal microbiota transplantation (FMT), which may provide new therapeutic options for complicated CDI in patients with UC [4-6]. This report draws attention to recurrent CDI as a persistent clinical challenge in elderly patients with UC and underscores the need for innovative strategies to improve management.

## Case presentation

We present a case of an elderly male (in his early 90s) with UC who was being treated with mesalazine and was brought to the hospital with severe hyponatraemia, delirium, and a fall. He was admitted for four months and had three separate episodes of CDI, all confirmed by stool studies. The CDI persisted through treatment with vancomycin, metronidazole, and fidaxomicin, and ultimately progressed to septic shock and death.

First CDI episode (September 20, 2024): The patient presented with loose stool, and stool assays confirmed CDI via stool PCR (in-house PCR from an MFT hospital laboratory). He was febrile at 38.2°C (Table 1). A stat dose of IV gentamicin 320 mg was administered, along with IV co-amoxiclav 1.2 g three times daily for an unknown infection source, likely urinary in origin. This was later stepped down to oral co-amoxiclav 500 mg/125 mg three times daily for a total of seven days. During this time, the patient was also started on oral vancomycin 125 mg four times daily. Following this treatment, symptoms showed minimal improvement.

**Table 1:** Investigations performed on September 20, 2024.

Test Name	Result	Out of Range	Reference Range
Neutrophils	16.86 × 10⁹/L	Yes	1.80-7.50 × 10⁹/L
WBC	20.5 × 10⁹/L	Yes	4.0-11.0 × 10⁹/L
CRP	101 mg/L	Yes	0-5 mg/L
*Clostridioides difficile* PCR	Positive	Yes	Negative

**Figure 1:**
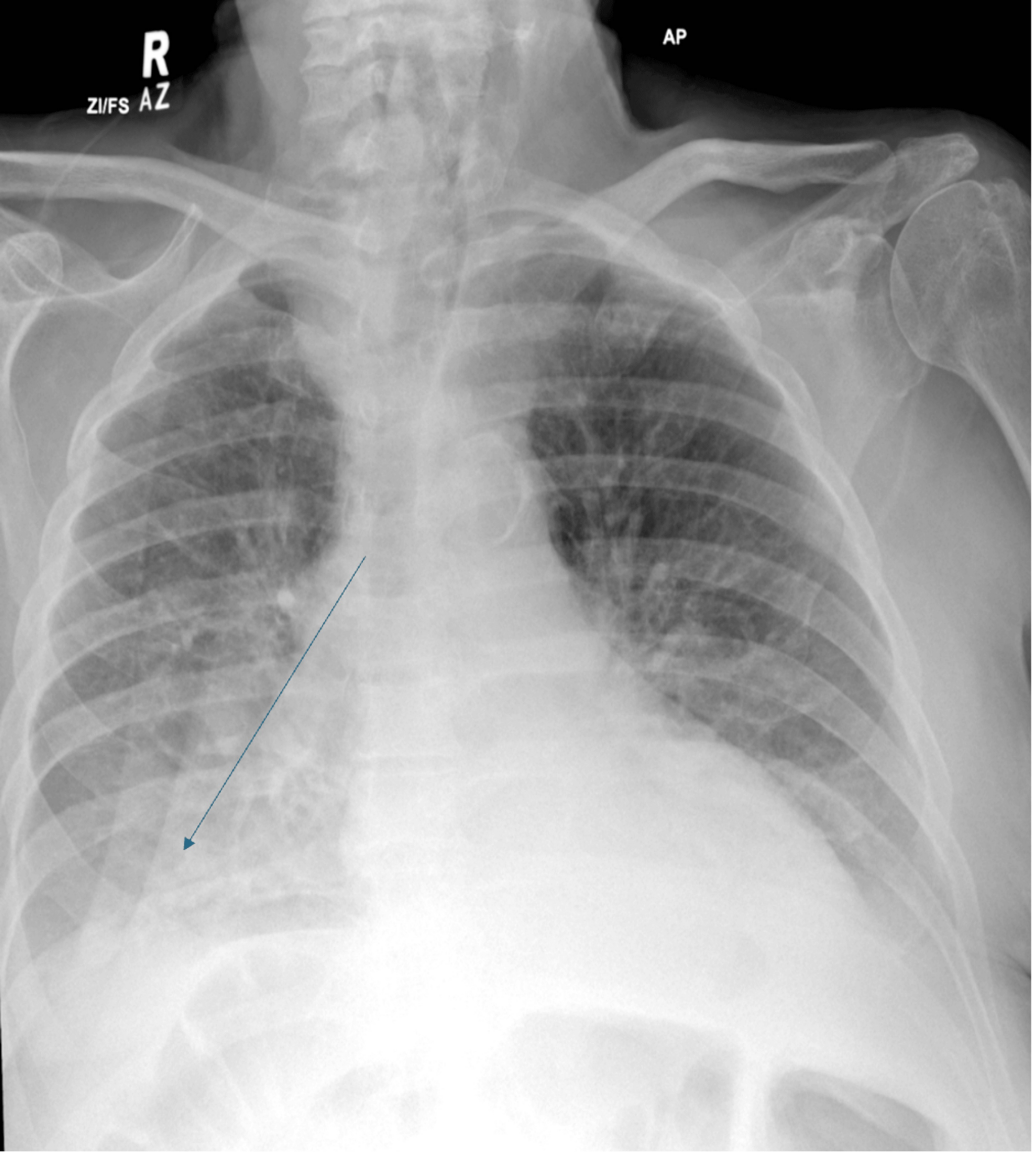
Chest X-ray taken on 24 October 2024 showing right-sided haziness and blunting of the costophrenic angle, suggestive of pneumonia. The patient was subsequently started on antibiotics on October 26, 2024.

Second CDI episode (October 20, 2024): The patient presented with several episodes of black, loose stool (types 6/7 on the Bristol Stool Chart). Again, stool assays confirmed CDI (Table 2). WBC count was 16.6 × 10⁹/L; lactate was 2.4 mmol/L; CRP increased from 115 mg/L (October 16) to 202 mg/L (October 20); temperature was 38.5°C; and BP was 99/51 mmHg (Table 3). He was treated with fidaxomicin 200 mg twice daily for a 10-day course. Faecal calprotectin was also significantly elevated at 1,373 µg/g. An important concurrent development was the onset of hospital-acquired pneumonia on October 26, based on clinical findings and chest X-ray (Figure 1). He was initially treated with IV co-amoxiclav, followed by escalation to tazocin for five days. It is worth noting that immunosuppressive therapy for UC was not escalated during this period.

**Table 2:** Investigations performed on October 20, 2024.

Test Name	Result	Out of Range	Reference Range
Faecal calprotectin	1,373 µg/g	Yes	0-100 µg/g
Neutrophils	14.10 × 10⁹/L	Yes	1.80-7.50 × 10⁹/L
WBC	16.6 × 10⁹/L	Yes	4.0-11.0 × 10⁹/L
CRP	202 mg/L	Yes	0-5 mg/L
*Clostridioides difficile* PCR	Positive	Yes	Negative

**Table 3:** Vital signs recorded on October 19, 2024.

Vital Sign	Value
Blood pressure	104/70 mmHg
Pulse	90 bpm
Temperature	38.0 °C (100.4 °F)
Respiratory rate	17 breaths/min
Height	1.89 m
Weight	60.1 kg
Oxygen saturation (SpO₂)	97%

**Figure 2:**
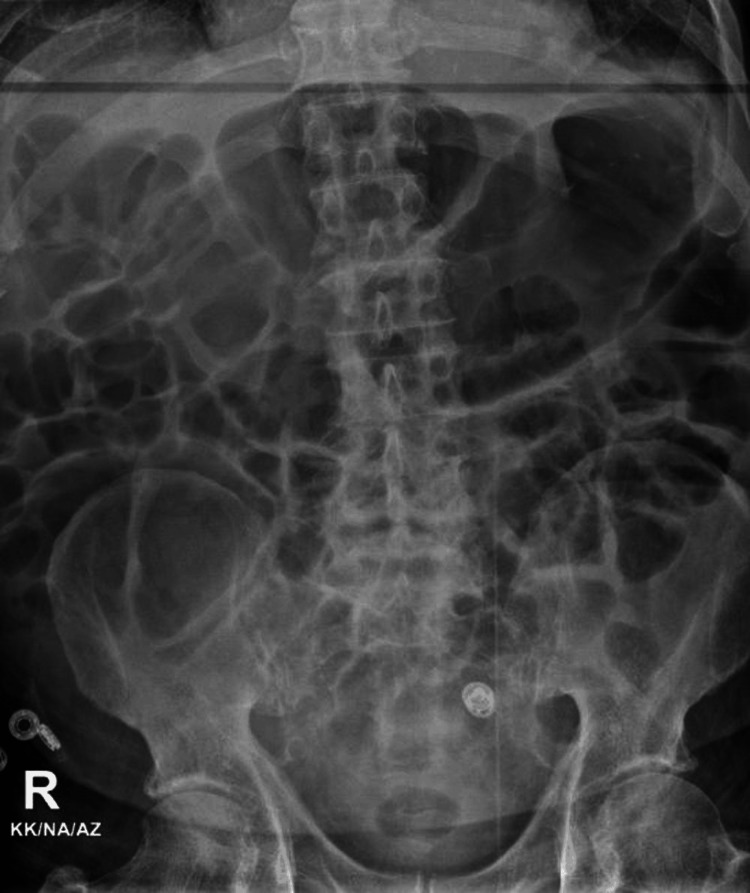
Abdominal X-ray showing a non-specific bowel gas pattern.

Third CDI episode (November 16-17, 2024): Despite ongoing treatment, the patient’s symptoms did not improve, and he experienced recurrent bouts of diarrhoea and melena. He was hypotensive (BP 74/42 mmHg), febrile (38.5°C), and had an elevated lactate of 2.7 mmol/L. Toxic megacolon was ruled out by abdominal X-ray (Figure 2). He rapidly deteriorated despite initiation of IV metronidazole 500 mg every eight hours and oral vancomycin 125 mg every six hours, and he passed away on November 17, 2024, likely due to Type 2 myocardial infarction (MI) secondary to sepsis.

## Discussion

This case highlights a subset of elderly patients with UC who are at greater risk and experience more complications from recurrent CDI. Distinguishing UC flares from CDI episodes is clinically challenging, in part due to overlap in symptomatology, including diarrhoea and abdominal pain [1-3]. An elevated faecal calprotectin level is indicative of inflammation in the colon but is not specific for differentiating CDI from exacerbations of UC [2,6]. This diagnostic overlap often leads to delays in appropriate therapy, emphasizing the importance of stool toxin assays or PCR testing in any UC patient experiencing worsening symptoms. The risk of CDI is higher in individuals with prior antibiotic exposure, particularly with broad-spectrum antibiotics, due to the disruption of balanced gut flora and subsequent colonization by pathogens [2,4]. Immunosuppressive therapy, often required in UC, further compounds this risk by altering host immune responses. Furthermore, frailty, comorbidities, and hospitalization are recognized predictors of recurrent CDI, which may partly explain the clinical course observed in this patient [7].

Vancomycin remains the current first-line treatment, with fidaxomicin as a second-line option. However, the recurrent nature of CDI in this patient indicates that alternative approaches are warranted [4,6]. The literature provides evidence that FMT yields favourable outcomes in treating recurrent CDI [8] and may be safely used in patients with recurrent CDI, particularly when standard antimicrobial therapy options are exhausted [4,6]. Another therapeutic option is bezlotoxumab, a human monoclonal antitoxin antibody that binds to *C. difficile* toxin B and neutralises its activity, thereby preventing recurrence of infection [9].

The findings of this case report align with previous literature [6]. This case underscores the critical importance of timely identification, appropriate stool-based diagnostics, and coordinated multidisciplinary care in older patients with UC who develop recurrent diarrhoea. For this vulnerable group, distinguishing CDI from a UC flare is essential to ensure prompt and effective management. It also emphasizes the need to consider advanced therapeutic strategies, such as faecal microbiota transplantation, bezlotoxumab, antimicrobial stewardship, and patient-specific risk assessment, to reduce both the occurrence and recurrence of CDI in individuals with underlying UC.

## Conclusions

This case demonstrates the significant risk and complexity of managing recurrent CDI in older patients with UC. Patients with UC are at an increased risk of developing recurrent CDIs, which pose substantial challenges in clinical management and are associated with higher mortality. Complications such as septic shock can arise even when an appropriate antibiotic regimen is prescribed. This reinforces the importance of close monitoring, judicious antibiotic stewardship, and consideration of alternative approaches, such as FMT, to help reduce morbidity and mortality.
